# Investigation of the Anticorrosive Activity of *Piper divaricatum* Essential Oil on Steel in 1 M HCl

**DOI:** 10.1021/acsomega.4c07269

**Published:** 2024-10-29

**Authors:** Heloise Leal Monteiro, Alcy Favacho Ribeiro, Mewry Joyce Correia Modesto, Eloisa Helena de Aguiar Andrade, José Antonio da Silva Souza, Jordan Del Nero, Arthur de Farias Silva Rente, Rodrigo Della Noce, José Pio Iúdice de Souza, Carlos Alberto Brito da Silva Jr, Marcos Vinícius da Silva Paula, Ana Aurea Barreto Maia

**Affiliations:** † Programa de Pós-Graduação em Ciência e Engenharia de Materiais, Universidade Federal do Pará, Ananindeua 67130-660, Brasil; ‡ Faculdade de Química, Universidade Federal do Pará, Ananindeua 67130-660, Brasil; § Museu Paraense Emílio Goeldi, Belém 66077-830, Brasil; ∥ Programa de Pós-Graduação em Engenharia de Recursos Naturais da Amazônia, Universidade Federal do Pará, Belém 66079-420, Brasil; ⊥ Faculdade de Física, Universidade Federal do Pará, Belém 66075-110, Brazil; # Faculdade de Química, Universidade Federal do Pará, Belém 66075-110, Brasil; ∇ Faculdade de Física, Universidade Federal do Pará, Ananindeua 67130-660, Brasil

## Abstract

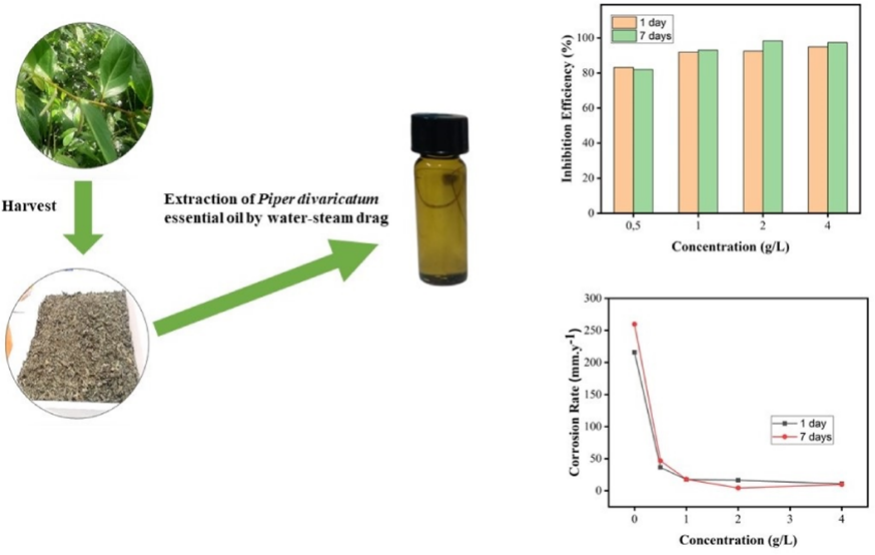

*Piper divaricatum* essential oil (PDEO), extracted from plants of the Brazilian Amazon, was investigated for the first time as a novel green or eco-friendly inhibitor for steel corrosion in 1 M HCl at 25 °C. Our electrochemical studies demonstrate that for different PDEO concentrations, lower *E*_corr_ and *i*_corr_ values were obtained. The influence of the oil concentration on corrosion inhibition, 0.5–4 g/L, was determined for 1 and 7 days of immersion. The corrosion rate (CR) and inhibition efficiency (IE) were determined by mass loss. The steel surface in the presence and absence of oil was investigated by scanning electron microscopy (SEM). The main PDEO compounds, determined by gas chromatography–mass spectrometry (GC-MS), were methyleugenol (20.68%) and eugenol (15.42%). The CR and IE for 2 g/L PDEO exhibited an optimal value of 4.31 mm/year and 98.3% for 7 days of immersion, respectively. The surface with 2 g/L oil for 7 days exhibited a less rough morphology, which was attributed to the corrosion inhibitory effect of PDEO. In addition, the PDEO adsorption process on the steel surface obeyed the Langmuir isotherm model. Negative values found for free standard energy ( < 0 kJ·mol^–1^) were attributed as a favorable process, i.e., an indicative of physisorption and chemisorption between the PDEO components and the steel surface. Our results reveal that the PDEO has a promising character for anticorrosive steel applications and metal coating in industries.

## 1 Introduction

Corrosion control of metals, alloys, and steel is of technical, economical, environmental, and aesthetical importance. Corrosion is a constant and continuous problem that is often difficult to eliminate completely. Prevention is more practical and achievable than complete elimination. The use of inhibitors is one of the best options for protecting metals, alloys, and steel against corrosion.^1^

Steel corrosion is a degradation process caused by oxidation,^2^ an aqueous or acidic medium on the steel surface. This phenomenon reduces the useful life of artifacts manufactured from steel. Acid media are widely used by industries in steel artifacts in situations such as pickling, oil well acidizing, and acid cleaning process.^3^

Steel corrosion in an acid medium can be inhibited with the use of organic inhibitors^4^ that act directly on electrochemical reactions, which are capable of delaying anodic, cathodic, or both activities.^5^ The 1 M HCl^5−7^ is very investigated in studies of natural corrosion inhibitors. 1 M H_3_PO_4_^8^ and 1 M H_2_SO_4_^9^ solutions were also investigated.

According to a study from *NACE* International, in 2016, about International Measures of Prevention, Application, and Economics of Corrosion Technologies (IMPACT) to examine the current role of corrosion management in industry and government and to establish best practices, the global cost of corrosion was estimated at $2.5 trillion (USD) per year, which is equivalent to 3.4% of the global Gross Domestic Product (GDP).^10^ Today, it is estimated to be around $3 trillion, which is almost 3.5% of the 2020 world GDP. Thus, there has been a search for new methods and initiatives to reduce costs associated with corrosion, with an increase in research for new alternatives of inhibitors, mainly by those from plant origin.^1^

Many inorganic and synthetic compounds have good anticorrosive activity, but they are highly toxic to human beings and the environment, often expensive, and nonbiodegradable.^11^ The environmental toxicity of organic corrosion inhibitors has prompted the search for corrosion inhibitors with low environmental impact and nontoxic properties made possible the use of green or natural corrosion inhibitors which are biodegradable, do not contain heavy metals, are usually obtained from plants, and have low cost.^12^ The traditionally used natural organic corrosion inhibitors in aqueous solutions are based on leaf extract, juice, and essential oil (EO).^7,8,13^

Thus, the use of natural products as corrosion inhibitors has become a key area of research because plant extracts are viewed as an incredibly rich source of naturally synthesized chemical compounds that are biodegradable in nature and can be extracted by simple procedures with low cost.^14^

The natural products extracted from plants (leaves, peels, seeds, fruits, and roots) have been widely studied as corrosion inhibitors of C-steel in acidic media.

Plants are green corrosion inhibitors, inexpensive, readily available, and renewable, as well as considered an inexhaustible source of environmentally friendly compounds whose heteroatoms (oxygen, sulfur, and nitrogen) and their π-electrons interact with the metallic surface through adsorption, mitigating the steel corrosive process,^15^ which reduces the corrosion rate (CR) and improves the inhibition efficiency (IE) in percentage (%).

Some oils of vegetable origin have characteristics of adsorbents, and this fact provides a protective layer on the metallic surface against corrosion. This is related to the presence of heteroatoms in the chemical compounds of the vegetable, such as oxygen, nitrogen, sulfur, and phosphorus.^1^

The recent exploitation of natural resources such as essential oils (EOs) from diverse plant sources as low-cost, green corrosion inhibitors is a promising area of research. Many EOs are used as potential inhibitors against the corrosion of iron or steel. The anticorrosion activity of EOs is normally attributed to the existence of complex organic components, such as oxygenated monoterpenes, sesquiterpenes, and hydrocarbons. These compounds generally contain polar groups with oxygen atoms and conjugated aromatic rings or double bonds. As such, these substances are susceptible to adsorbing on metal surfaces.^16^

*Artemisia mesatlantica* EO was proposed as a natural inhibitor for steel corrosion in 1 M HCl. In this study, a maximum IE of 92% was found using a concentration of 3.0 g/L.^17^ For cinnamaldehyde, obtained from cinnamon EO, an IE of 95.36% was found for 0.2 g/L of inhibitor.^16^ For 0.5 M HCl, clove EO exhibited an IE of 94% for steel using a concentration of 4 g/L. The authors reported that the anticorrosive activity occurred due to the association between the oil components.^18^ An IE of 85% for steel corrosion in a 1 M HCl medium was observed for the EO extracted from the leaves of *Dysphania ambrosioides*.^19^

The EO of the species *Piper divaricatum*, belonging to the Piperaceae family, which is the most abundant of the 12 families of plants in the Amazon region, is extracted from leaves by harvest via water-steam drag exhibiting biological activities (antioxidant, bacterial, fungicidal, and larvicidal activity) due to the diversity of chemical components and physicochemical characteristics present in this volatile oil, with emphasis on antioxidant activity. This property, when in ideal concentrations in a corrosive environment, considerably inhibits or delays oxidative processes.^20,21^

Corrosion is a spontaneous form of metal degradation or deterioration by a chemical, biochemical, or electrochemical process, in which the metal undergoes an oxidation reaction in the presence of some chemical medium or element that can be reduced, altering its chemical, physical, or mechanical properties until it reaches its lowest energy state.^22^ In nature, the vast majority of metals are in the form of oxides or hydroxides that are in the minimum energy state. Due to this trend, refined metals used in infrastructure, transportation, production, manufacturing, and electronic equipment when in contact with the environment tend to return to their natural or stable thermodynamic state, i.e., into a more chemically stable form such as oxide, hydroxide, or sulfide.^23^

Dhouibi et al. (2021) studied the anticorrosive effects of EOs of rosemary (REO) and myrtle (MEO), extracted by the Clevenger technique and analyzed using the gas chromatography–mass spectrometry (GC-MS), which are rich in various volatile compounds and act as cathodic-type green inhibitors for copper corrosion in a 3 wt% NaCl solution. The EO molecule adsorption on the copper surface followed a Langmuir isotherm, and physical adsorption (vs chemical adsorption) is dominant. The IE reached 91.88% and 92.54% at 10 g/L for MEO and REO, respectively.^24^ Ihamdane et al. (2023) studied the anticorrosive effect on the carbon steel surface of EO of oregano (*Origanum vulgare*) leaf in a 1 M HCl solution. The EO molecule adsorption on the carbon steel surface followed a Langmuir isotherm, by the formation of rigid covalent bonds. The IE reached 85.64% at 2 g/L for oregano in 1 M HCl.^25^ Already, Bathily et al. (2021) reported a review on EOs and their corrosion-inhibiting properties.^26^

In this work, the *Piper divaricatum* essential oil (PDEO), from the Brazilian Amazon, was investigated for the first time by mass loss experiments as a novel green corrosion inhibitor of steel in 1 M HCl at 25 °C for different concentrations (0, 0.5, 1.0, 2.0, and 4.0 g/L) and days (1 and 7). The PDEO was extracted by the water-steam drag technique using a Clevenger-type glass system, and its chemical composition was determined by GC-MS. Gravimetric measurements were carried out in this study and enabled to determine the inhibition efficiency (IE) and corrosion rate (CR) of this oil, its mode of action, as well as certain parameters specific to corrosion. The surface morphology was observed by scanning electron microscope (SEM) images, and the Langmuir adsorption isotherm was obtained to analyze the adsorption mechanism on the metallic surface. The aim is to expand studies on the evaluation of the PDEO with the potential for inhibiting corrosion, carrying out experimental and theoretical tests in order to contribute to research on plant anticorrosive inhibitors, reinforcing their use on industrial scales and their advantages and benefits for the environment, in contrast to the toxicity of traditionally used organic and inorganic inhibitors. This investigation contributes to the proposal of a new natural inhibitor of corrosion in an acidic environment and presents the biomass available for the extraction of EO as the main challenge.

## 2 Experiment

### 2.1 Materials

The materials used in this investigation are listed in Table 1.

**Table 1:** Materials, Description, and Supplier Used in the Preparation of the PDEO

Material	Description	Supplier
Hydrochloric acid	37%, PA, molecular weight 36.46 g/mol	Êxodo científica
Acetic acid	Pure, molecular weight 60.05 g/mol	Êxodo científica
Sodium sulfate	Molecular weight 322.20 g/mol	Êxodo científica
Galvanized steel sheet	Galvanic-coated steel sheets	Aço Cearense

### 2.2 Methodology

#### 2.2.1 Extraction of PDEO

**Figure 1:**
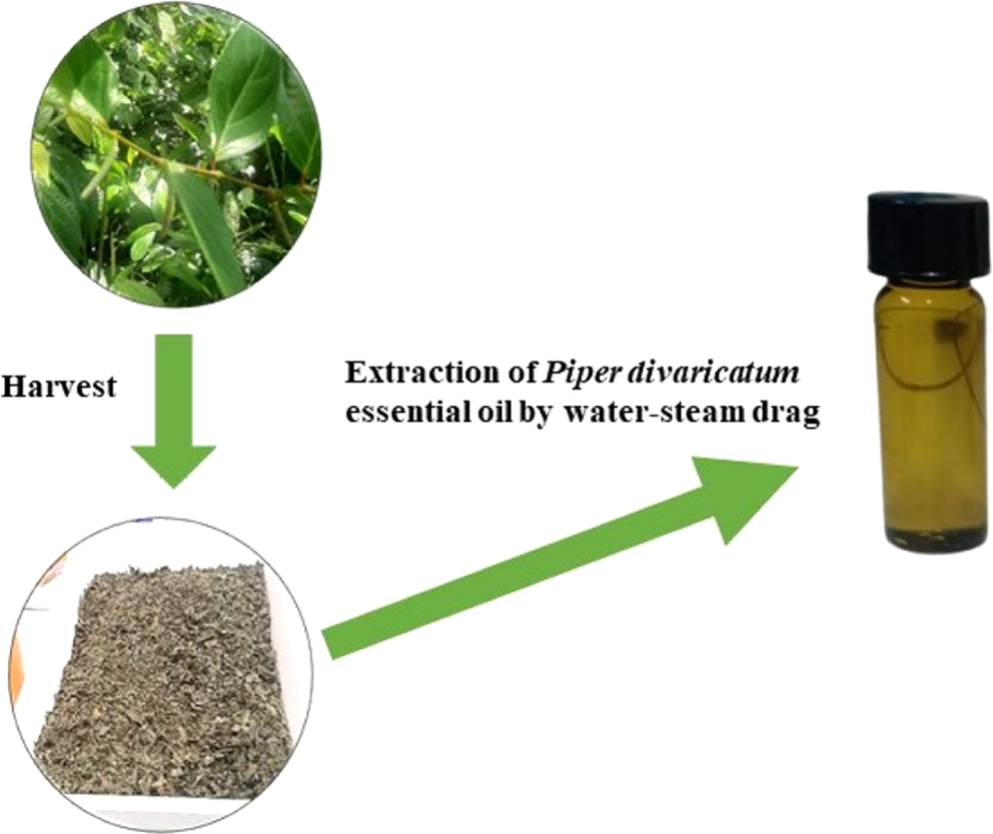
Extraction of PDEO.

The essential oil was extracted from the leaves of *Piper divaricatum*, from plants collected in the city of Belém, Brazil, by water-steam drag using a Clevenger-type glass system. The botanical material was collected at Cidade Universitária “José Silveira Netto” (Latitude: 1° 27′ 48.5″ S, Longitude: 48° 26′ 38.8″ W). Leaves and thin branches were selected, and then, the plant material was placed in an oven with air circulation at 35 °C. After this stage, the dry material was crushed in a knife mill and homogenized. The extraction system used Clevenger-type glass equipment, coupled to a refrigeration system to maintain condensation water between 10 and 15 °C for 3 h. After extraction, the oil was centrifuged in a Pinmax 80–2B system for 5 min at 3000 rpm, dehydrated with anhydrous Na_2_SO_4_, and centrifuged again under the same conditions described (Figure 1). The chemical composition of the oil was determined by gas chromatography–mass spectrometry (GC-MS), using a Shimadzu Model QP 2010 Ultra instrument, equipped with an Rtx-5MS fused silica capillary column (30 m × 0.25 mm; 0.25 μm film thickness).

#### 2.2.2 Electrochemical Studies

Polarization curves for steel samples with and without PDEO in 1 M HCl were acquired using an Autolab model PPGSTAT302 potentiostat/galvanostat instrument. The instrument was equipped with three electrode cells: working (steel sample), reference (silver and silver nitrate), and counter electrode (platinum). The potential was determined in the range of −0.550 to −0.150 V.

#### 2.2.3 Mass Loss Experiments

Coupons were cut from a galvanized steel sheet supplied by Aço Cearense. They had dimensions of 2 cm × 2 cm and also an orifice with a 5 mm diameter. The coupons were degalvanized with 10% acetic acid from Êxodo Cientfica within 24 h. After that, the coupons were washed with distilled water until the total removal of acetic acid residues and dried at 80 °C for 30 min. For the gravimetric tests, the coupons were weighed and immersed in 100 mL of 1 M HCl with PDEO, 0.5–4 g/L, for 1 and 7 days of immersion. The tests were performed in triplicate for each concentration of oil used. After 1 and 7 days, the coupons were removed, washed with distilled water, dried, and weighed. The inhibition efficiency (IE) in percentage (%) was calculated by eq 1:^16^
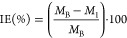
1

where *M*_B_ and *M*_1_ are the coupon mass losses without and with the inhibitor, respectively. The corrosion rate (CR) was calculated by eq 2:^13^
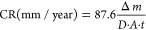
2

where Δ*m* = difference in coupon mass (mg) before and after immersion in the HCl solution, *D* = metal density (∼7.8 g/cm^3^), *A* = coupon area (cm^2^), and *t* = time (h).

#### 2.2.4 Adsorption Isotherm Models

The adsorption isotherms were obtained to analyze the adsorption mechanism on the metallic surface as shown in eqs 3^6^. The correlation coefficient (*R*^2^) was used to determine which isotherms of Langmuir, Temkin, Frumkin, and Freundlich are most suitable for the adsorption of oil onto the steel surface. To determine the isotherms, graphs were obtained based on the following isotherm models:^16,27,28^
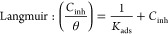
3
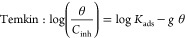
4
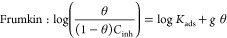
5
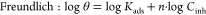
6

where *C*_inh_ = inhibitor concentration (g/L), θ = degree of surface coverage, *K*_ads_ = constant of the adsorption–desorption reaction (or distribution coefficient), *g* is an adsorbate interaction parameter, and *n* is a correction factor, but θ is calculated by eq 7.^16^
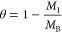
7

The standard free energy of adsorption () is given in kJ·mol^–1^ by eq 8:^29,30^
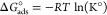
8

where *R* is the universal gas constant (8.314 J·mol^–1^·K^–1^), *T* is the temperature (K), and K° is the standard equilibrium constant (dimensionless).

#### 2.2.5 Surface Analysis

SEM images were obtained using a TESCAN electronic microscope, model Mira3. The images were generated by the detection of secondary electrons with a voltage acceleration of 15 kV and a distance of 15 mm.

## 3 Results and Discussion

### 3.1 Oil Composition

Table 2 exhibits the constituents found for PDEO. The presence of 53 constituents was observed, where the main components are sesquiterpene hydrocarbons and phenylpropanoids with levels of 52.87% and 38.41%, respectively. Korkina reports that the phenylpropanoid class exhibits antioxidant, anti-inflammatory, antiviral, healing, and antibiotic properties;^31^ and sesquiterpenes found in genus *Carpesium* plants are important for the aroma of natural products and have a series of pharmacological properties, such as antitumor, anti-inflammatory, antibacterial, antiparasitic, insecticidal, and antiviral activities.^32^

**Table 2:** Identification of Chemical Constituents Present in PDEO^a^

RI_L_	RI_C_	Constituent	(%)
932	930	α-Pinene	0.44
974	933	β-Pinene	0.46
988	977	Myrcene	0.04
1024	990	Limonene	0.07
1044	1028	(*E*)-β-Ocimene	0.53
1135	1046	*trans-*Pinocarveol	0.04
1137	1138	*trans-*Sabinol	0.05
1194	1144	Myrtenol	0.07
1285	1196	Safrole	0.88
1335	1289	δ-elemene	3.33
1345	1330	α-Cubebene	0.26
**1356**	1366	**Eugenol**	**15.42**
1374	1382	α-Copaene	1.00
1389	1390	β-Elemene	10.20
**1403**	**1419**	**Methyleugenol**	**20.68**
1417	1436	(*E*)-Caryophyllene	6.35
1430	1444	β**-**Copaene	1.85
1439	1452	Aromadendrene	1.19
1447^*^	1456	Isogermacrene D	0.39
1452	1465	α-Humulene	1.74
1478	1473	γ-Muurolene	0.52
1484	1485	Germacrene D	10.41
1489	1501	β-Selinene	1.81
1500^*^	1513	Biciclogermacrene	8.34
1511	1516	δ-Amorphene	0.27
1513	1524	γ-Cadinene	1.67
1520	1528	α-*epi-*Selinene	0.10
1522	1534	δ-Cadinene	3.01
1524	1537	Chavibetol Acetate	1.43
1533	1541	*trans*-Cadina-1,4-diene	0.26
1537	1545	α-Cadinene	0.13
1544	1550	α-Calacorene	0.04
1548	1556	Elemol	0.02
1561	1571	(*E*)-Nerolidol	0.68
1577	1589	Spatulenol	1.88
1582	1594	Caryophyllene Oxide	1.34
1592	1600	Viridifloren	0.07
1600	1609	Rosifoliol	0.07
1608	1617	Humulene Epoxide II	0.12
1618	1620	1,10-*diepi*-Cubenol	0.06
1629	1629	Eremoligenol	0.02
1627	1634	*epi*-Cubenol	0.05
1645	1638	Cubenol	0.04
1639	1644	*allo*-Aromadendrene Epoxide	0.12
1640	1648	α-*epi*-Murrolol	0.24
1644	1652	α-Murrolol	0.11
1652	1662	α-Cadinol	0.51
1668	1665	(*E*)-9-*epi*-14- Caryophyllene Hidroxide	0.12
1685	1676	Germacra-4, (15), 5, 10, (14)-trien-1-α–ol	0.17
1685	1689	α-Bisabolol	0.03
1687	1692	Eudesma-4, 15-dien-1-β-ol	0.04
1745	1723	γ-Costol	0.03
1845^*^	1845	2-Pentadecanone	0.07
Monoterpene Hydrocarbon			1.54
Oxygenated Monoterpenes			0.16
Sesquiterpene Hydrocarbons			52.87
Sesquiterpene Oxygenated			5.79
Phenylpropanoids			38.41
Total			98.77%

a RI_L_: literature retention index;^41^ RI_C_: calculated retention index; * retention index identified by the FFNSC-2 database.

The chemical composition of PDEO was characterized by the presence of the following: (1) phenylpropanoids: methyleugenol (20.68%) and eugenol (15.42%) and (2) sesquiterpene hydrocarbons: germacrene D (10.41%), β-elemene (10.20%), bicyclogermacrene (8.34%), and (*E*)-caryophyllene (6.35%). These components are frequently identified in essential oils from *Piper divaricatum*, collected in the western region of the state of Pará, Brazil.^33^ Oliveira et al. identified the presence of methyleugenol and eugenol in high levels in the essential oil of the dried leaves of the species *Piper divaricatum*, collected in the city of Belém in the state of Pará.^34^ Eugenol (or 4-allyl-2-methoxyphenol) is widely used in pharmacological, gastronomic, and medicinal areas. This compound has antioxidant, antimicrobial, fungicidal, and anti-inflammatory properties.^35^ Methyleugenol (or 4-allyl-1,2-dimethoxybenzene) is used in industries of hygiene products, adhesive products, cosmetics, perfumes, shampoos, and soaps.^36^

Montanari et al. observed that for essential oils from the species *Aloysia virgata*, *Lippia brasiliensis*, *Lantana montevidensis*, and *Lantana trifolia*, Germacrene D was the main component. In addition, the essential oils exhibited fungicidal and bactericidal activity.^37^ β-elemene has antitumor and anti-inflammatory activities and is used medicinally via oral or injectable liposome emulsion for the treatment of some types of cancer in China. However, few studies prove the effectiveness of this compound. This constituent is often extracted from the *Curcuma wenyujin* plant and used in traditional Chinese medicine.^38,39^ Haldhar et al. studied three phytochemicals, such as eugenol, methyleugenol, and cinnamyl acetate, that are excellent inhibitors for the corrosion of carbon steel due to their molecular structures and their occurrence in a variety of plants.^40^ Thus, the main constituents found in PDEO indicate that this oil has a high potential to inhibit metallic corrosion.

### 3.2 Electrochemical Studies

**Figure 2:**
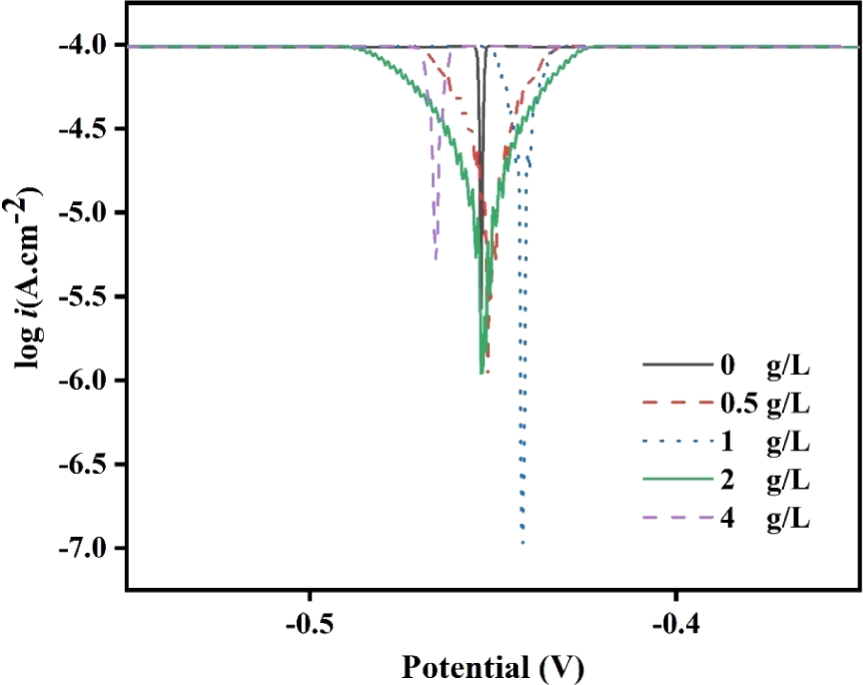
Polarization curves for steel in 1 M HCl after 24 h of immersion with different concentrations of PDEO.

Polarization curves for steel with different concentrations of PDEO, 0.5–4 g/L, are provided graphically in Figure 2. Additionally, polarization parameters such as corrosion potential (*E*_corr_), current density (*i*_corr_), cathodic Tafel slope (β_c_), and anodic Tafel slope (β_a_) are listed in Table 3. Our findings demonstrate that for different PDEO concentrations, lower *E*_corr_ and *i*_corr_ values were obtained. The polarization curves display the predominance of inhibition for cathodic reactions; the shape of the curves in the cathodic region exhibited smaller changes compared to that same region of the curve without PDEO.^27,52^ On the other hand, βa values exhibited a significant modification, which indicates the effect of PDEO on the formation of iron ions.^27,52^ However, the cathode displacement with 0.5–4 g/L of PDEO is less than 0.085 V, which prevents classifying PDEO as a cathodic or anodic inhibitor.^52^ Thus, our results show that PDEO is a mixed-type corrosion inhibitor (cathodic/anodic).^27,52^ The addition of PDEO promoted an inhibition of steel corrosion in 1 M HCl.

**Table 3:** *E*_corr_, *i*_corr_, −β_c_, and β_a_ for PDEO in 1 M HCl Medium, after 24 h of Immersion

Concentration (g/L)	*E*_corr_ (V)	*i*_corr_ (A/cm^2^)	–β_c_ (Vdec^–1^)	β_a_ (Vdec^–1^)
0	–0.455	2.841 × 10^–5^	0.017	0.004
0.5	–0.451	6.894 × 10^–6^	0.019	0.019
1	–0.442	1.758 × 10^–5^	0.022	0.02
2	–0.453	9.198 × 10^–6^	0.037	0.04
4	–0.465	1.129 × 10^–5^	0.007	0.01

### 3.3 Gravimetric Measurement

**Figure 3:**
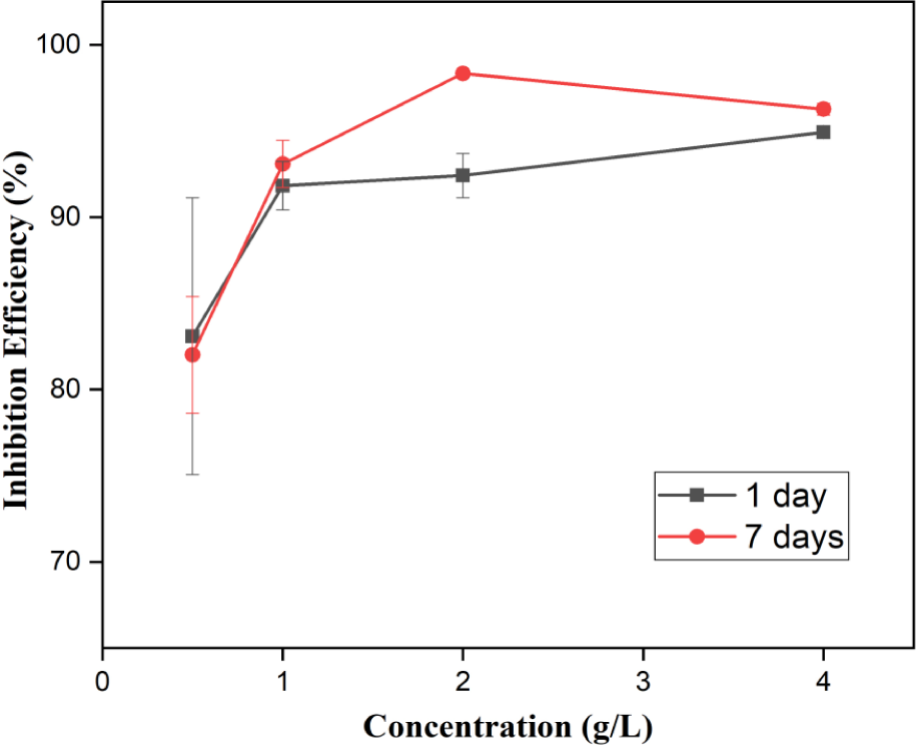
IE of PDEO for 1 and 7 days.

**Figure 4:**
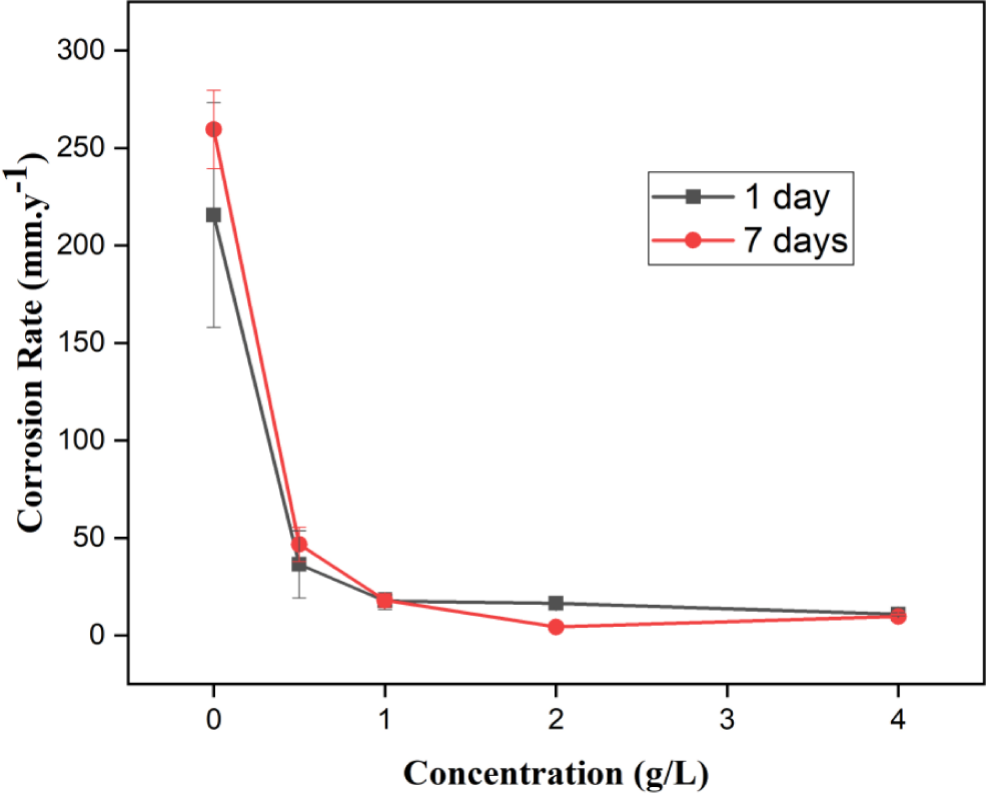
CR of PDEO for 1 and 7 days.

The investigation of PDEO as a promoter of corrosion inhibition of steel in 1 M HCl was carried out by mass loss tests for 1 and 7 days, with oil concentrations ranging from 0 to 4 g/L. Table 4 and Figures 3, 4 summarize the results for IE, CR, and θ on steel in an acid medium with PDEO. All errors or uncertainties in the calculation of IE and CR are exhibited in Table 4 although some error bars do not appear in Figures 3,4 because they are very small. In general, it was observed that increasing the concentration of the EO led to an increase in the IE value for the two immersion periods evaluated.

**Table 4:** IE, CR, and θ for PDEO in 1 M HCl Medium

Concentration (g/L)	Inhibition efficiency (%)	Corrosion rate (mm·y^–1^)	θ
0 (1 day)	-	215.71 ± 57.66	-
0.5 (1 day)	83.09 ± 8.03	36.46 ± 17.31	0.8309
1 (1 day)	91.83 ± 1.40	17.60 ± 4.35	0.9183
2 (1 day)	92.42 ± 1.27	16.35 ± 2.75	0.9242
4 (1 day)	94.93 ± 0.34	10.92 ± 0.73	0.9493
0 (7 days)	-	259.63 ± 20.13	-
0.5 (7 days)	82.02 ± 3.38	46.69 ± 8.79	0.8202
1 (7 days)	93.09 ± 1.36	17.93 ± 3.54	0.9309
2 (7 days)	**98.33** ± 0.11	**4.31** ± 0.30	0.9833
4 (7 days)	96.27 ± 0.34	9.67 ± 0.88	0.9627

Figure 3 for the IE values reveals that the PDEO performed satisfactorily as a green corrosion inhibitor for steel and the increase in IE was proportional to the increase in PDEO concentration, where it is observed that the highest concentration provides greater corrosion inhibitory activity. The highest IE value equal to 94.93% was obtained for an oil concentration of 4 g/L for 1 day, i.e., oil concentrations greater than 1 g/L exhibit marginal variations in the IE values for 1 day. The IE found for 2 g/L PDEO was remarkably equal to 98.33% for 7 days of immersion. This result confirms that the PDEO with a concentration of 2 g/L acted successfully in inhibiting steel corrosion in 1 M HCl. This concentration (2 g/L) is considered satisfactory because it corresponds to the smallest amount of oil used that provides the highest IE.

Rizi et al. extracted and characterized an essential oil (EO) obtained from the *Cuminum cyminum* (CC) plant native to India to act as a corrosion inhibitor on mild steel in a 0.5 M HCl solution at different concentrations. They found that the *Cuminum cyminum* L. (CCL) extract effectively reduces the corrosion of mild steel in hydrochloric acid with an inhibition efficiency ranging from 79.69% to 98.76%. The optimal inhibition concentration was 2 g/L of EO, similar to ours, and surface analysis confirmed the formation of a protective layer. Furthermore, our results suggest that the inhibitor binds to the metal surface through a charge-transfer process, creating a protective film.^42^

Figure 4 shows that the CR has an inverse behavior to that determined for the IE for 1 and 7 days. The effect of oil addition was inversely proportional to the CR, and the progressive increase in the oil concentration decreased the CR value. For steel immersed in 100 mL of 1 M HCl without oil for 1 day, a CR of 215.71 mm·y^–1^ was found, while with 1 g/L of the oil, a decrease in that of 17.60 mm·y^–1^ was obtained. The CR from the oil concentration of 1 g/L was observed stability for the CR for 1 day of immersion. This behavior is in good agreement with the value calculated for the IE for this concentration, where a plateau in the IE was observed of inhibition from the concentration of 1 g/L. For 7 days of immersion, the highest value of CR was 4.31 mm·y^–1^ for an oil concentration of 2 g/L. This concentration of 2 g/L for PDEO is a satisfactory concentration to promote the inhibition of steel corrosion in a 1 M HCl medium.

The anticorrosive activity of PDEO was attributed to the adsorption of its main components onto the steel surface, which is favored by the low pH of the corrosive environment. The oil components adsorbed on the steel surface prevent the occurrence of the characteristic reactions observed in the corrosive process in an acid medium^16,43^ (see the “Surface Investigation” section). Our results are corroborated by Loto, where, by potentiometric method, a steel corrosion inhibition efficiency value of 97.69% (0.5 M HCl) was obtained for a mixture formed by rosemary essential oil and *Cinnamon cassia* essential oil.^44^ Hossain et al. used cinnamaldehyde extracted from cinnamon essential oil as a green inhibitor for steel corrosion in a 10% HCl medium. They observed via mass loss experiments an inhibition efficiency of 95.36% for a concentration of 0.2 g/L of cinnamaldehyde. The inhibitory activity of cinnamaldehyde was attributed to its adsorption on the steel surface, minimizing the action of the corrosive environment.^16^ Growcock and Frenier, using *trans*-cinnamaldehyde, found by mass loss tests an inhibition efficiency for steel corrosion in 15% HCl of 91.9%.^45^ Our findings are in good agreement with several studies that investigated natural, green, or eco-friendly inhibitors for metal (steel, copper, etc.) corrosion in acidic medium such as caffeine,^46^ cinnamon essential oil,^47^*Syzygium aromaticum* aqueous extract,^2^*Dysphania ambrosioides* essential oil,^19^*Origanum vulgare* essential oil,^25^*Thunbergia fragrans* extract,^48^ and *Eucalyptus globulus* essential oil.^49^

### 3.4 Surface Investigation

**Figure 5:**
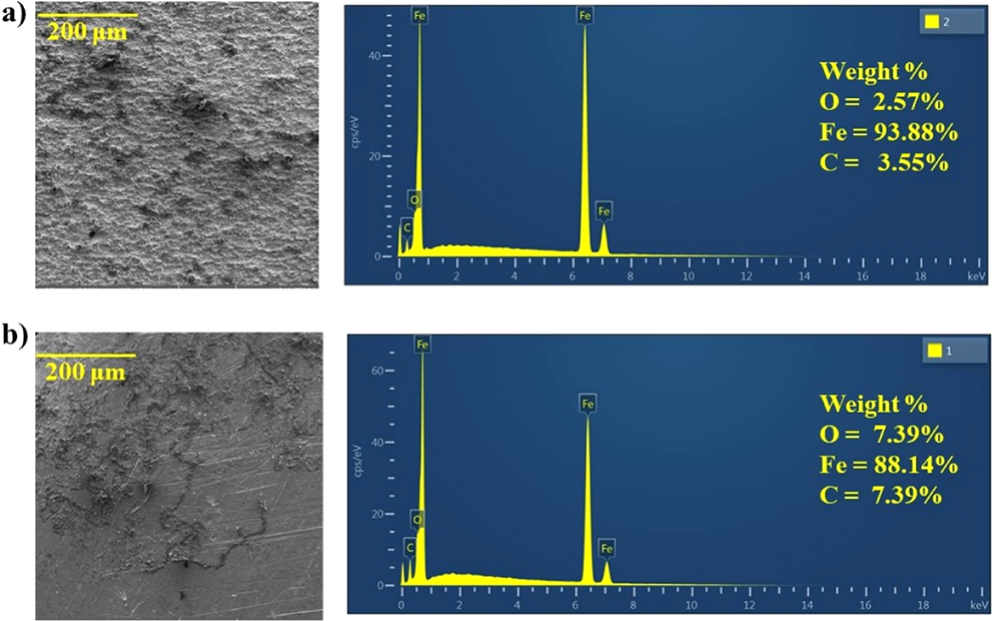
Surface morphologies (on the left) and EDS (on the right) for steel: (a) without inhibitor and (b) with inhibitor for 7 days.

Figure 5 exhibits the steel surface immersed in 1 M HCl (a) without and (b) with PDEO through the acquisition of SEM images. Figure 5a shows that the surface of steel without oil is rough, which occurs in the presence of a corrosive acid medium. The surface with 2 g/L of oil for 7 days exhibited a less rough morphology. This result is attributed to the corrosion inhibitory effect of PDEO (Figure 5b). Energy-dispersive spectroscopy (EDS) showed that the steel surface is composed mainly of iron, carbon, and oxygen. EDS also revealed that carbon is in a higher amount on the steel surface with the oil, which suggests that the oil components are adsorbed on the steel surface and also causes a decrease in the iron content. In addition, EDS showed that the iron content of steel in an acid medium is lower in the presence of PDEO. This behavior can be explained by the adsorption of PDEO components on the steel surface, and similar results were reported by Sanaei et al.^15^

### 3.5 Adsorption Isotherm

**Figure 6:**
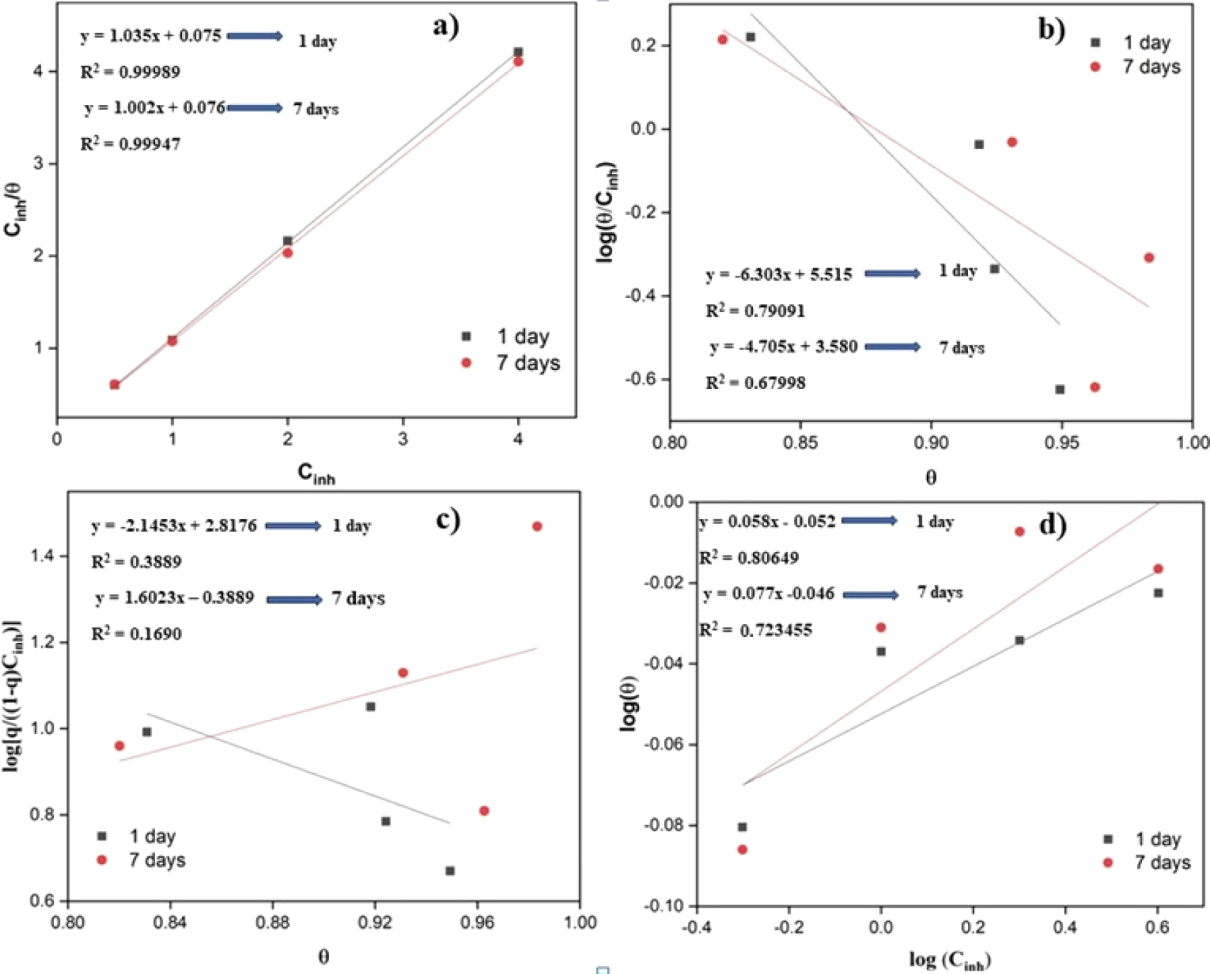
Isotherm models for the adsorption of PDEO on the steel surface in 1 M HCl for 1 and 7 days: (a) Langmuir, (b) Temkin, (c) Frumkin, and (d) Freundlich.

The isothermal adsorption models of Langmuir, Frumkin, Temkin, and Freundlich were evaluated (black and red lines) and compared with experimental results (black closed squares and red closed circles) to explain the mode of adsorption of the PDEO components on the steel surface in a 1 M HCl medium for 1 and 7 days, respectively. Figure 6a–d displays the graphs for the four investigated models. Figure 6a shows that the graph of *C*_inh_/θ versus *C*_inh_ is a straight line, which is satisfactorily attributed to the Langmuir adsorption isotherm model, with values of *R*^2^ and a slope very close to 1 (Table 4).

These results indicate that there is a monolayer of oil components adsorbed on the steel surface^16^. Sanni, Iwarere, and Daramola reported that the Langmuir adsorption isotherm model was found to describe the adsorption behavior of the parsley essential oil to inhibit the corrosion of aluminum alloys in simulated seawater with 3.5% NaCl solutions.^50^ For *Dysphania ambrosioides* essential oil, thermodynamic studies found that the adsorption of oil components on the steel surface in 1 M HCl obeys the Langmuir model.^19^ Cristofari et al. also found that the adsorption of *Helichrysum italicum* subsp. *italicum* essential oil components on steel in a 1 M HCl medium also obeys the Langmuir adsorption model.^51^ On the other hand, for *Peganum harmala* seed extract, as a natural inhibitor for steel, the Freundlich adsorption isotherm model was determined.^52^ Date palm leaf extract also follows the Freundlich adsorption isotherm model on the aluminum surface in an acidic medium.^53^ Graphs for the Temkin, Frumkin, and Freundlich isotherms displayed *R*^2^ and slope values significantly lower than 1, which means that the PDEO does not obey these adsorption isotherm models (Figure 6b–d).

PDEO has several components (see the “Oil Composition” section), and some components are in higher content. Thus, our mechanistic proposal for corrosion inhibition by PDEO will be demonstrated for two major components (eugenol and methyleugenol). However, this mechanism is also applicable to the minority components, which also have lone pairs of electrons, heteroatoms, and aromatic rings. In the literature, there are several adsorption studies where *K*_ads_ is dimensional. However, for the standard free energy calculation (eq 8), the K° is dimensionless. Thus, considering the exchange adsorption, the value of *K*_ads_ (L·g^–1^) needs to be corrected by eq 9.^54^
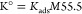
9

where *M* = molecular weight of eugenol (164.2 g·mol^–1^) or methyleugenol (178.2 g·mol^–1^).

Table 5 displays, as an example, a negative value for  for the adsorption of eugenol in 1 and 7 days, which means that the adsorption process is a favorable process. These standard free energy calculations can be performed for the other components of the PDEO. Thus,  values less than −40 kJ·mol^–1^ correspond to chemisorption, while  values higher than −20 kJ·mol^–1^ are attributed to physisorption.^27^ The values for  are close to −30 kJ·mol^–1^, which is an indicative of both physisorption and chemisorption between the oil components and the steel surface. High values for *K*_ads_ indicate efficient adsorption, which promotes high inhibition efficiency (IE) as observed for 2 g/L in 7 days.^43^ Lashgari et al. reported a high dispersion for *K*_ads_ for *Thymus vulgaris* leaf extract as an effective mild-steel corrosion retardant for 200, 400, 600, and 800 ppm of extract at different immersion times.^28^ A similar trend was found for *Tamarindus indica* aqueous extract as a new green inhibitor for mild steel in an acidic medium.^43^

**Table 5:** Langmuir Adsorption Isotherm Parameters (Eugenol)

Concentration (g/L)	Time (days)	*K*_ads_ (L·g^–1^)		(kJ·mol^–1^)
**0.5**	1	12.7 ± 9.1	115610	–28.9
	7	9.3 ± 1.9	85374	–28.1
**1**	1	11.7 ± 3.0	106973	–28.7
	7	13.9 ± 3.3	126821	–29.1
**2**	1	6.2 ± 1.2	56756	–27.1
	7	29.7 ± 2.1	270442	–31.0
**4**	1	4.7 ± 0.3	42821	–26.4
	7	6.4 ± 0.6	59199	–27.2

**Figure 7:**
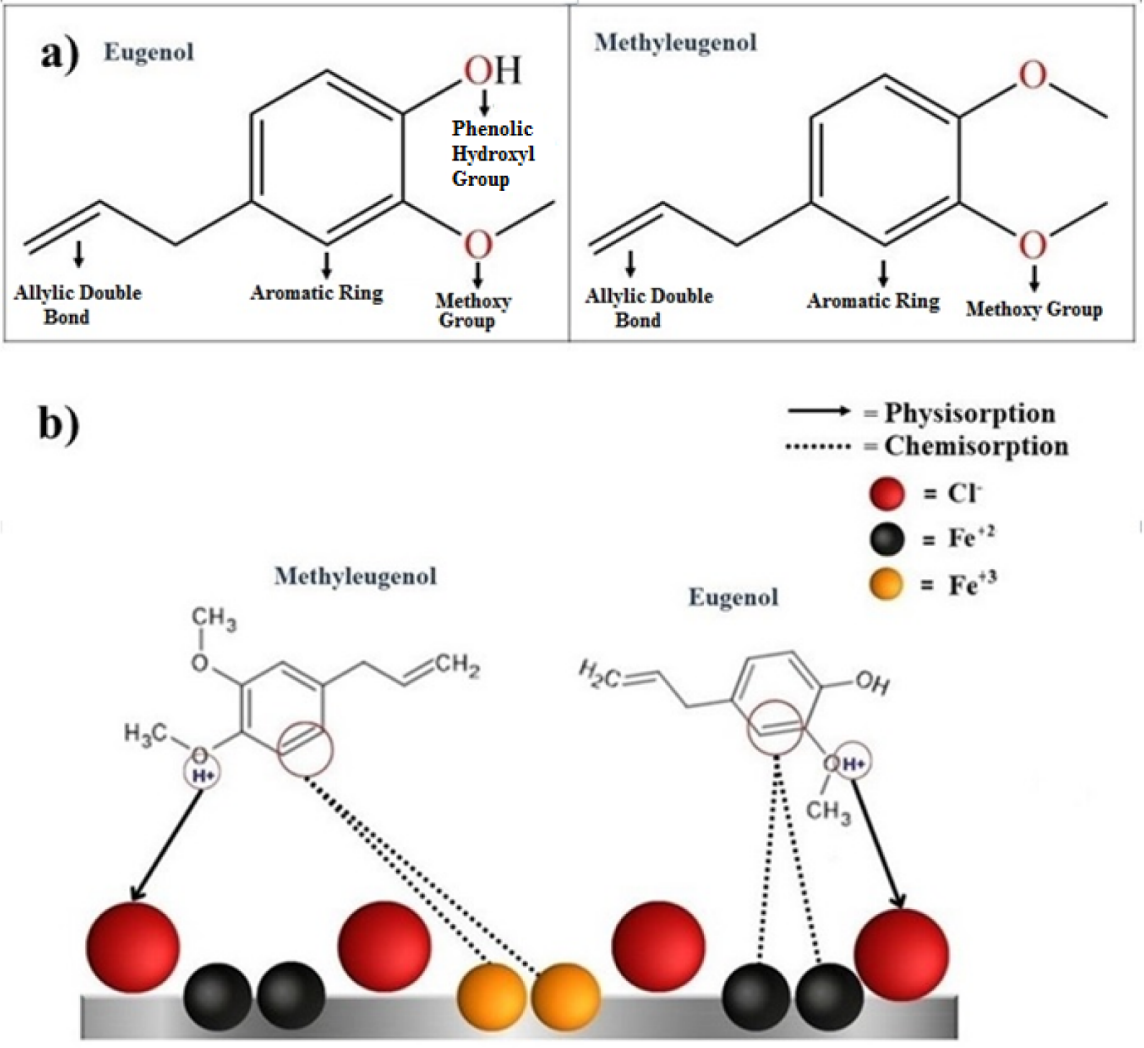
(a) Chemical structure of eugenol (C_10_H_12_O_2_), methyleugenol (C_11_H_14_O_2_), and their reaction sites. (b) Mechanism of inhibition of steel corrosion (Fe) in acidic media (HCl) by PDEO.

The surface of steel in an acidic medium (HCl) has a positive charge (H^+^) which interacts electrostatically with Cl^–^ anions (red balls in Figure 7b). The molecules of the main components of PDEO can be protonated through oxygen (O) atoms, which are in red in Figure 7a.^55^ These protonated molecules are electrostatically attracted by chloride anions (Cl^–^), and the adsorption process occurs by physisorption (Figure 7b). For chemisorption, lone electron pairs and heteroatoms of PDEO components can interact chemically by donating electrons to iron ions (Fe^2+^ and Fe^3+^, see black and yellow balls in Figure 7b).^28^ Similar mechanistic proposals have been reported for the *Thymus vulgaris* leaf extract as a green corrosion inhibitor.^28^ This proposed mechanism for inhibiting steel corrosion by PDEO is supported by our experimental IE and CR results, where the main components of PDEO (determined by GC-MS) were eugenol (15.42%) and methyleugenol (20.68%; Table 2). Thus, our results reveal that PDEO, a natural product, extracted in the Brazilian Amazon, inhibited the corrosion of steel in 1 M HCl with an inhibition efficiency of 98% for a concentration of 2 g/L of PDEO.

## 4 Conclusion

The PDEO was studied as a green corrosion inhibitor for steel in an acid medium. From the obtained results, we have the following conclusions: GC-MS analysis found eugenol and methyleugenol as the main components of PDEO. Our electrochemical studies demonstrate that for different PDEO concentrations, lower *E*_corr_ and *i*_corr_ values were obtained. The experimental and theoretical data show that PDEO acts as an effective inhibitor of steel corrosion in 1 M HCl. The maximum value for steel corrosion IE was equal to 98.33% for an oil concentration of 2 g/L for 7 days of immersion. Corrosion rate (CR) values decreased as the concentration of PDEO increased. The corrosion process was inhibited by the adsorption of organic matter on the steel surface. The steel surface with PDEO exhibited less damage than the steel surface without PDEO. The oil adsorption process of PDEO on the steel surface from 1 M HCl obeys the Langmuir adsorption isotherm. Negative values found for  indicate the adsorption as a favorable process, and the adsorption mechanism is typical of physisorption and chemisorption. Based on the characterization of PDEO, the major components (eugenol and methyleugenol) act together by adsorption (physisorption and chemisorption) to ensure inhibition. Then, the inhibition is regarded as an intermolecular synergistic effect of the various components of natural oil or essential oil. These findings reveal that the PDEO, extracted in the Brazilian Amazon, has a promising character for anticorrosive steel applications and for metal coating in industries, which is in good agreement with the literature.
